# Adaptation of a brief intervention to support engagement in early psychosis services: a service user-informed psychotherapy modification and study protocol

**DOI:** 10.3389/fpsyg.2026.1763354

**Published:** 2026-09-22

**Authors:** Brannon Senger, Lilith Nosko, Alissa Pencer, Candice E. Crocker, Debbie Emberly, Philip G. Tibbo

**Affiliations:** 1 Department of Psychology and Neuroscience, Dalhousie University, Halifax, NS, Canada; 2 Department of Psychiatry, Dalhousie University, Halifax, NS, Canada; 3 Mental Health and Addictions, IWK Health, Halifax, NS, Canada; 4 Department of Diagnostic Radiology, Dalhousie University, Halifax, NS, Canada

**Keywords:** early intervention in psychosis, early phase psychosis, engagement in treatment, psychotherapy adaptation, psychotherapy trial, study protocol

## Abstract

Early intervention services (EIS) for psychosis are associated with improved clinical and functional outcomes, yet disengagement from treatment remains common. Psychological barriers to early engagement such as stigma, mistrust, and negative treatment expectancies have been identified yet few interventions have been developed to directly target these. *Just Do You* is a brief, psychotherapeutic meta-intervention originally developed for youth with severe mental illness to target identified barriers to engagement and has been demonstrated to improve engagement and recovery. However, the context of the original trial differs from Canadian early psychosis programs in setting, clinical population characteristics, and timing of delivery, necessitating adaptation for suitability in this context. This project comprises two phases. Phase 1, now complete and reported in this manuscript, used the Formative Method for Adapting Psychotherapy (FMAP) and the Psychotherapy Adaptation and Modification Framework (PAMF) to adapt *Just Do You* for a Canadian EIS. A multidisciplinary stakeholder group, including service users, participated in structured consultation meetings to guide the adaptation process. Their feedback aligned with four key domains: strengthening trust and autonomy, enhancing relevance to early psychosis, improving feasibility of delivery, and supporting continuity beyond sessions. Resulting modifications and the adapted intervention are described in depth in this manuscript. Phase 2 of this project, aims to evaluate the adapted intervention using a non-randomized wait-list control design. This manuscript also outlines the Phase 2 research protocol, including planned assessment procedures and analytic strategies. Findings from Phase 2 will inform future large-scale evaluation of the adapted intervention.

**Clinical trial registration:**

ClinicalTrials.gov, Identifier NCT06410508.

## Introduction

Early intervention treatment for psychosis has become the standard of care for individuals experiencing early phase psychosis (EPP). Multiple well-designed research trials and meta-analyses have demonstrated that early intervention care leads to improved outcomes compared to treatment as usual: Those who access early intervention services (EIS), show improvements in psychopathology (Correll et al., 2018; Kane et al., 2016; Nolin et al., 2016; Norman et al., 2011), quality of life (Kane et al., 2016), functional outcomes (Bond et al., 2015; Correll et al., 2018; Kane et al., 2016), lower hospitalization rate (Correll et al., 2018; Kane et al., 2016), decreased risk of suicide and suicide attempt (Tahmazov et al., 2025), and are less likely to disengage from treatment (Correll et al., 2018; Kane et al., 2016). These benefits are particularly robust when services are accessed early, and individuals remain highly engaged throughout the duration of care (Kane et al., 2016). Engagement is therefore a key determinant of whether the benefits of EIS are realised. Yet the psychological barriers that impede early engagement, though well described, have rarely been targeted directly by intervention, and no engagement-focused intervention has been developed or adapted for a Canadian early psychosis context.

These clinical benefits are accompanied by evidence for the cost-effectiveness of EIS. A systematic review of economic evaluations concluded that EIS is likely to be cost-effective across a range of health systems, though the authors note substantial heterogeneity in the underlying studies (Aceituno et al., 2019). More recent modelling work has estimated EIS to be cost-saving from a societal perspective and cost-effective from a health system perspective, driven in part by reductions in hospitalization (Sediqzadah et al., 2022), with similar conclusions reported in a Canadian evaluation (Tarride et al., 2022). The economic case for EIS, however, rests on service users receiving the treatment these programs provide, making sustained engagement a determinant of both clinical and economic returns. Despite this considerable promise, barriers to optimal outcomes persist.

A substantial body of research has examined factors associated with outcomes and engagement in EIS, and these can be grouped broadly into three categories. The first comprises sociodemographic and clinical characteristics, including age at onset (Immonen et al., 2017; Schimmelmann et al., 2008), duration of untreated psychosis (Díaz-Caneja et al., 2015; Stowkowy et al., 2012; Weiss et al., 2021), substance use (Golay et al., 2020; Seddon et al., 2016), socioeconomic status (Hatzimanolis et al., 2020), racial or ethnic identity (Maraj et al., 2018a; Waldron et al., 2022), and migrant status (Maraj et al., 2018b; Ouellet-Plamondon et al., 2015). These variables have been associated with both recovery trajectory and service engagement and may operate directly as markers of illness severity or indirectly by influencing help-seeking and continued involvement in care. The second comprises relational and service-level factors, including therapeutic alliance and the availability of individual psychotherapy (Polillo et al., 2022) and family involvement in treatment (Claxton et al., 2017; Mascayano et al., 2021; Revier et al., 2015; Robson and Greenwood, 2022), which are potentially modifiable but have received comparatively less attention. The third, and least examined, comprises experiential factors: how individuals come to access services and the quality of their prior encounters with the mental health system. Notably, systematic reviews have identified few consistent predictors of engagement beyond substance use, family involvement, forensic involvement, and medication adherence (Mascayano et al., 2021; Robson and Greenwood, 2022), suggesting that the variables studied to date account only partially for who remains engaged in care.

Engagement in treatment is a key predictor of outcomes in EIS. Unsurprisingly, the therapeutic benefits of EIS are most fully realized when individuals participate meaningfully and access the full continuum and course of available care (Doyle et al., 2014; Kane et al., 2016). Despite this, available research suggests disengagement from EIS, although less than in usual treatment, remains unacceptably high; on average, approximately 30% of individuals who initially engage in EIS disengage at some point during the course of their treatment (Doyle et al., 2014; Mascayano et al., 2021; Polillo et al., 2022). A recent systematic review of 20 studies found disengagement rates ranging from 12 to 53% (Mascayano et al., 2021), while another found engagement rates to be around 16% (Robson and Greenwood, 2022) with variability likely being due in part to the varied way engagement is defined and measured. While some studies use binary definitions (e.g., absence from services for three consecutive months), others apply continuous metrics (e.g., number of missed appointments) or rely on self-report surveys of service involvement (Doyle et al., 2014; Mascayano et al., 2021; Polillo et al., 2022). Beyond appointment attendance, the quality of individuals’ interactions with services, including trust and openness with providers and perceived usefulness of care may represent critical but underexamined indicators of engagement (Munson et al., 2020, 2022).

For many individuals, the pathway from initial help-seeking to accessing EIS is not easily navigated (Anderson et al., 2013; Gannon et al., 2023; Jones et al., 2021; MacDonald et al., 2021; Senger et al., 2024, 2025). In many contexts, referrals are most made through emergency departments or inpatient psychiatric units, often following multiple failed help-seeking attempts. These referrals often occur during acute illness, and a significant proportion involve involuntary hospitalization (Gannon et al., 2023; Jones et al., 2021; Senger et al., 2024, 2025). Prior research has suggested that, when accounting for psychotic symptom severity, individuals referred through inpatient or emergency departments are more likely to need future acute psychiatric intervention such as hospitalization during their time in EIS (Senger et al., 2024). Moreover, previous research has found that those with referral experiences involving emergency services are less likely to attend their initial consultation with EIS, and these individuals, as well as those with law enforcement involvement during referral, are more likely to disengage from EIS over time (Polillo et al., 2022; Robson and Greenwood, 2022; Weiss et al., 2021).

Qualitative research also supports these findings. Service users who have been hospitalized, particularly involuntarily, prior to accessing EIS often describe these experiences as frightening, coercive, or punitive, and many report entering EIS with a sense of hopelessness or mistrust towards mental health services (Jones et al., 2021; Senger et al., 2025; Silva et al., 2023; van Schalkwyk et al., 2015). When describing aspects of their referral experiences that shaped negative perceptions of EIS, service users most often cited a loss of autonomy, lack of transparent communication about treatment plan, threats to physical safety, and dismissive attitudes from healthcare professionals. However, even among those not referred through coercive pathways, early skepticism or ambivalence about treatment is common (Senger et al., 2025). These insights suggest that, while individuals with prior negative experiences may be at heightened risk for disengagement, many young people accessing EIS for the first time may benefit from support aimed at strengthening engagement early in care.

Existing engagement interventions have largely focused on reducing external barriers to care by reducing cost or providing reminders and transportation for appointments (Kim et al., 2012). While valuable, these approaches do not address the internal psychological barriers to engagement such as stigma, negative beliefs about providers, hopelessness about recovery, and reduced sense of self-agency or self-efficacy (Munson et al., 2020, 2022). Addressing these barriers requires an intervention that targets beliefs and expectancies about treatment rather than access to it. To be feasible in an early psychosis setting, such an intervention would also need to be brief, deliverable alongside routine care, and offered early enough to reach individuals during the period when disengagement risk is greatest.

One promising approach is a brief “meta-intervention” developed by Munson and colleagues for young adults with serious and persistent mental illness (SPMI), called *Just Do You* (Munson et al., 2020, 2022; Rodwin et al., 2024). Informed by cognitive-behavioral principles and grounded in the *Young Adult Service Use Framework*, this intervention targets common psychological barriers to engagement, such as self-stigma, mistrust, negative expectancies about treatment, and hopelessness. Delivered collaboratively by a peer support worker and clinician, *Just Do You* was shown to increase service engagement and self-reported recovery outcomes in its original trial, primarily by fostering stronger buy-in to treatment (Munson et al., 2020, 2022).

Although originally developed for a general SPMI population, *Just Do You* targets psychological barriers to engagement that are also common among young people experiencing early psychosis, suggesting its potential relevance for this group (Munson et al., 2020, 2022; Rodwin et al., 2024; Senger et al., 2025). However, important differences exist between the original trial context and other settings. Compared to the Canadian EPP context, the intervention was initially delivered in a large urban U. S. setting with a general SPMI youth sample (ages 15–25), whereas EPP populations may have larger proportions of older patients (ages 12–35), are more diagnostically homogeneous, and treated within a single-payer, universally funded healthcare system (Munson et al., 2020, 2022; Senger et al., 2024). Additionally, delivering the intervention within the first 4 weeks of care, as done in the original trial, may not be feasible in EPP settings, where many clients are not yet prepared to engage in psychotherapy so early in their treatment trajectory. These differences suggest that an adaptation of the original *Just Do You* manual is necessary for successful implementation in early intervention for psychosis services. The current project aims to adapt the *Just Do You* engagement intervention for use within a Canadian early psychosis program in Nova Scotia and to evaluate its feasibility and preliminary effectiveness.

Overall, this project consists of two phases outlined in this manuscript: Phase 1, Therapy Adaptation, *now complete*, focused on engaging patient partners in a structured adaptation of *Just Do You* to enhance the relevance and acceptability of the intervention for a Canadian EIS context. This manuscript presents the outcomes of Phase 1, detailing the adaptation procedures and the resulting modifications to the therapy manual. Phase 2: Evaluation, involves implementing the adapted *Just Do You* intervention and assessing its effects on self-reported engagement, recovery, internalized stigma, insight, and other relevant clinical indicators using a non-randomized wait-list control design. This manuscript provides a description of the research protocol guiding Phase 2 of the project.

These two phases are reported together for two reasons. First, established methods for adapting psychotherapy treat empirical testing of the revised protocol as an integral phase of adaptation rather than a separate undertaking; the adaptation reported here should therefore be understood as provisional pending evaluation. Second, several features of the Phase 2 evaluation protocol, including the selection of outcome measures and the parameters of intervention delivery, follow directly from Phase 1 adaptation decisions and are difficult to interpret in isolation. Based on findings from the original *Just Do You* trial, we hypothesize that (1) participation in the adapted intervention will be associated with improvements in treatment engagement, personal recovery, and insight as well as reductions in internalized stigma; and (2) participants who received the intervention would show fewer missed appointments, emergency department visits, and hospitalizations compared to controls. Once Phase 2 is complete, findings from this project are anticipated to inform a larger-scale implementation and evaluation of the adapted *Just Do You* intervention.

## Methods

Note on trial status: this protocol was submitted in December 2025, prior to completion of Phase 2. Data collection has since concluded, with results to be reported separately. The protocol and analysis plan described here were pre-specified and are reported as submitted.

### Project participants and setting

This project is being conducted at the Nova Scotia Early Psychosis Program (NSEPP), a specialized early intervention service located in Halifax, Nova Scotia, Canada. NSEPP provides care to individuals aged 12 to 35 with a recent or suspected diagnosis of a primary psychotic disorder and serves a catchment area of approximately 500,000 people. The NSEPP provides comprehensive wraparound care for individuals experiencing psychosis, including lowest-effective-dose pharmacotherapy, evidence-based psychotherapy (including CBT for psychosis), and a range of psychosocial interventions such as psychoeducation, family and peer support, and vocational and educational assistance. The program has an open referral system, and referrals are accepted from a range of sources including self-referral, family members, and healthcare providers. The clinic receives between 100 and 150 referrals annually, with 55–90 of these meeting criteria for acceptance to the program. Diagnoses are confirmed through comprehensive intake assessments conducted by licensed clinicians using DSM-5 criteria. Individuals with psychosis secondary to a medical condition, affective psychosis, or intellectual disability are excluded from participation. This study received approval from the Nova Scotia Health Authority Research Ethics Board (File no. 1030617).

### The *Just Do You* intervention

*Just Do You* is a brief, two-session engagement-focused meta-intervention, designed to address common barriers to engagement (e.g., stigma, mistrust, hopelessness) using elements of CBT and Motivational Interviewing (Munson et al., 2020, 2022). The intervention is co-delivered by a clinician and a peer support worker (PSW) with lived experience of psychosis who provides recovery-oriented support through shared understanding (Munson et al., 2020, 2022). The original intervention was delivered in a group format over two sessions, each lasting approximately 90 to 120 min and delivered within the first four to 6 weeks of treatment. The first module emphasized recovery-oriented thinking, challenged stigma and hopelessness, and explored ways of building trust with providers. The second module incorporated creative tools such as music and visual art to support emotional expression, process difficult past experiences with the healthcare system, and help participants identify goals for recovery. For additional detail on the original intervention, see Munson et al. (2020, 2022).

## Phase 1: adaptation of *Just Do You*

### Adaptation framework

The adaptation process was guided by two complementary frameworks developed by Hwang to support the modification of existing psychotherapies for diverse populations: the *Formative Method for Adapting Psychotherapy* (FMAP) and the *Psychotherapy Adaptation and Modification Framework* (PAMF) (Hwang, 2006, 2009). The FMAP is a community-based, “bottom-up” participatory approach involving active consultation with service users comprised of five iterative phases: (a) generating knowledge about potential adaptations through stakeholder collaboration; (b) integrating this knowledge with existing research, theory, and clinical understanding to identify and inform necessary adaptations; (c) reviewing the preliminary adapted intervention with stakeholders and incorporating their feedback into revisions; (d) empirically testing the revised protocol version; and (e) Finalizing the adapted intervention (Hwang, 2009).

In contrast, The PAMF offers a more theory-driven, “top-down” approach using a three-tiered structure: identifying broad domains for cultural consideration, outlining specific adaptation principles, and providing theoretical rationales for why those adaptations enhance therapeutic relevance and effectiveness (Hwang, 2006). Hwang recommends using these frameworks in tandem to ensure adaptations are both empirically grounded and acceptable to the target population (Hwang, 2009). In practice, the PAMF complements the FMAP by ensuring that theoretical, research, and clinical evidence are systematically integrated when reviewing stakeholder feedback, particularly prior to beginning stakeholder consultations and during FMAP phases *b* and *c*, when researchers synthesize stakeholder input with existing literature to develop and refine proposed adaptations in consultation with stakeholders. Our team used the FMAP’s participatory structure to guide engagement in adaptation meetings and consensus building with stakeholders, while the PAMF informed how we reviewed evidence and theory to evaluate the clinical and conceptual soundness of proposed changes.

Consistent with the PAMF, existing research and theory were reviewed at the outset to identify potential areas for adaptation prior to stakeholder consultation. The original *Just Do You* intervention established theoretical and empirical foundations for addressing engagement challenges among youth with serious mental illness (Munson et al., 2020, 2022). Our team’s previous qualitative research at NSEPP identified similar facilitators and barriers to engagement among young adults in EIS, as well as context-specific needs such as fostering autonomy and providing greater choice within sessions to build trust and engagement (Senger et al., 2025). Additional literature outlining principles for adapting psychotherapy for individuals with psychosis (Lakeman, 2006; Langman-Levy et al., 2023; Lecomte et al., 2017) was also reviewed. Together, this theoretical and empirical background provided a rationale for the need to adapt the intervention, offered initial guidance on areas where adaptations might be relevant, and served as a reference point during adaptation to ensure that stakeholder-informed modifications remained consistent with existing empirical and theoretical understanding.

### *Just Do You* adaptation process

As part of phase *a* of the FMAP, a multidisciplinary focus group was convened to discuss adapting the original *Just Do You* protocol. Seven individuals participated in the consultation meetings: a licensed clinical psychologist with expertise in early psychosis, a doctoral trainee in clinical psychology, a master’s-level research student, and four individuals with lived experience of psychosis. Of those with lived experience, one was a peer support worker (PSW) at the clinic who would later co-lead therapy sessions, and three were service users currently receiving care at NSEPP. The process involved three structured consultation meetings (each 2–3 h) and supplementary review of revised materials between meetings.

Consultation began with an orientation to the project background, rationale, and objectives, followed by an introduction to the adaptation frameworks. Stakeholders were invited to share their initial impressions of the study, reflect on early treatment experiences, and offer ideas for fostering safety and engagement during the initial stages of care. Subsequently, under the facilitation of a research team member (BS, doctoral student and trainee psychologist), stakeholders engaged in a detailed review of the original *Just Do You* intervention manual and provided structured feedback on content, session format, and delivery. Discussions were open-ended, with care taken not to influence service users’ perceptions of the material or potential adaptations. Conversations focused on the suitability of existing content, identification of gaps, and suggestions for modification to enhance relevance for individuals early in treatment for psychosis. Detailed minutes were taken during meetings on all feedback and recommendations. One researcher (BS) facilitated discussion while a second (LN) recorded structured minutes organised by intervention component, documenting stakeholder comments, research team responses, and proposed adaptations. Minutes were circulated to the research team and to stakeholders after each meeting, with stakeholders invited to correct any account they considered inaccurate.

Consistent with FMAP Phase *b*, the research team reviewed stakeholder feedback to identify the areas and nature of needed adaptations (Hwang, 2009). Guided by the PAMF (Hwang, 2006), proposed modifications were considered alongside existing literature on psychotherapy adaptations for psychosis populations (Lakeman, 2006; Langman-Levy et al., 2023; Lecomte et al., 2017). Using this information, the team developed provisional revisions to the *Just Do You* protocol, which were discussed at subsequent consultation meetings.

Consistent with FMAP Phase *c*, potential adaptations were then iteratively reviewed and revised with stakeholders. Proposed adaptations were documented as written summaries describing each proposed change and its rationale, and returned to stakeholders for review, with opportunity to endorse, revise, or object to each proposed change. This process was iterative: modifications were revised and re-circulated across meetings and between them until stakeholders either endorsed the adapted materials or raised no further concerns on subsequent review. Disagreement among stakeholders was uncommon, and where it arose it was resolved through discussion, with flexibility built into the protocol where preferences diverged. For example, differing views on the acceptability of group delivery early in treatment informed the decision to offer the intervention in either group or individual format. The adapted intervention was piloted with the PSW to assess feasibility and flow, after which stakeholders provided final feedback before the provisional manual was finalized.

## Phase 2: evaluation of the adapted *Just Do You* intervention

### Study design and procedures

Phase 2 aims to evaluate the preliminary effectiveness of the adapted *Just Do You* intervention when delivered within a Canadian EIS. A prospective, non-randomized repeated-measures wait-list control design will be used, enabling all participants to receive the intervention while still allowing for between-group comparisons. This design was selected because prior trials have demonstrated benefit of *Just Do You* (Munson et al., 2022) and withholding a potentially engagement-enhancing intervention from at-risk young people raises ethical concerns (Street and Luoma, 2002).

Participants will be recruited from the Nova Scotia Early Psychosis Program (NSEPP) and will be eligible if they: (1) are between ages 19 and 35; (2) are within their first 3–12 months of EIS treatment; and (3) are competent in written and spoken English. The three-month lower limit reflects clinical consultation during Phase 1, in which clinicians and stakeholders raised concern that many clients may not yet be ready to engage in psychotherapy during the initial weeks of care, consistent with concerns regarding the feasibility of the original trial’s four-week delivery window. As disengagement risk remains elevated across the first year of treatment (Doyle et al., 2014; Lal and Malla, 2015), this window allows the intervention to be delivered as early as is feasible while remaining within the period of greatest risk. Participants will be approached as soon as they become eligible. Initial participants will be allocated to the experimental condition (adapted *Just Do You* + treatment as usual). Once recruitment for this group is complete, subsequent eligible participants will be assigned to the wait-list control condition (treatment as usual, followed by the adapted intervention after the initial comparison period).

### Outcome measures and assessment timeline

Phase 2 of this project focuses on assessing whether the adapted intervention improves early treatment engagement and related psychological factors that are theorized to influence service engagement and downstream clinical outcomes for individuals with early psychosis. Consistent with the design of the original *Just Do You* trial, participants in both study conditions will complete assessments at four time points: baseline, 2 weeks post-enrollment (corresponding to after completion of Session 1 for the experimental group), 4 weeks post-enrollment (corresponding to after completion of Session 2), and a three-month follow-up (Munson et al., 2020, 2022).

At baseline, in addition to clinical assessments outlined below, participants will complete demographic assessments, including the collection of postal code data for the derivation of socioeconomic status using the Canadian Index of Multiple Deprivation (CIMD). The CIMD provides validated census-based indicators of residential instability, economic dependency, ethno-cultural composition, and situational vulnerability (Matheson et al., 2012; Pampalon et al., 2012), enabling examination of social determinants that may influence engagement in early psychosis services.

Primary outcomes to be measured include self-reported engagement in treatment, personal recovery, internalized stigma, and insight into illness. Engagement and personal recovery were selected to allow comparison with the original *Just Do You* trial, where both were evaluated as primary outcomes and demonstrated improvement (Munson et al., 2020, 2022). Engagement will be assessed using a modified version of the Yatchmenoff Client Engagement Scale, which measures treatment receptivity, buy-in, therapeutic alliance, and trust (Yatchmenoff, 2005). Personal recovery will be measured using the Recovery Assessment Scale–Revised (RAS-R), a widely used instrument assessing confidence and hope, help-seeking, symptom management, reliance on others, and goal orientation (Corrigan et al., 2004; Salzer and Brusilovskiy, 2014).

Internalized stigma and insight were added as primary outcomes in the present study as stigma was a target of the original *Just Do You* intervention but was not measured in the initial trial. Both constructs are also believed to be associated with engagement in early psychosis services. Internalized stigma will be assessed using the 9-item Internalized Stigma of Mental Illness Scale (ISMI-9) (Boyd et al., 2014). Insight will be measured using the VAGUS Self-Report Scale, which evaluates illness awareness, symptom attribution, and perceived need for treatment (Gerretsen et al., 2014).

Secondary outcomes will include clinician-rated indicators of symptomatology and social and occupational functioning. These domains are being measured to allow for evaluation of the association between clinical and functional outcomes and primary outcomes engagement and to examine whether the adapted Just Do You intervention is associated changes in symptom severity or psycho-social functioning. Psychotic symptom severity will be assessed using the PANSS-6, a validated brief version of the Positive and Negative Syndrome Scale (Kay et al., 1987; Østergaard et al., 2016). Global illness severity will be rated using the Clinical Global Impression–Severity and Improvement scales (CGI-S, CGI-I) (Guy, 1976). Functioning will be evaluated using the Social and Occupational Functioning Assessment Scale (SOFAS), which assesses role functioning independent of symptom severity (Morosini et al., 2000). Substance use will be assessed using the WHO Alcohol, Smoking, and Substance Involvement Screening Test (ASSIST) and relevant modules of the Structured Clinical Interview for DSM-5 (SCID-5), with a focus on alcohol and cannabis given their prevalence and association with engagement in EIS (First et al., 2015; WHO ASSIST Working Group, 2002; Hides et al., 2009). Trauma/Adversity history, will be assessed using the Trauma and Life Events (TALE) checklist (Carr et al., 2018), enabling examination of whether prior adversity or coercive pathways to care predict intervention effects on engagement.

### Administrative health outcomes

To complement self-reported and clinician-rated data, administrative health records will be used to capture objective indicators of service involvement and acute care utilization. Psychiatric hospitalizations, mental-health-related emergency department visits, and missed outpatient appointments at NSEPP will be extracted for the 3 months preceding participants’ enrollment and the 3 months following participation. Missed appointments are included as a direct, objective indicator of engagement in treatment, while hospital and emergency department data allow examination of whether changes in engagement correspond to reduced acute service use. Consistent with previous research, exploratory subgroup analyses will also examine differences based on referral pathway (urgent vs. non-urgent), as pathways involving emergency or coercive services have been associated with mistrust, ambivalence, and heightened risk of disengagement (Jones et al., 2021; Senger et al., 2025; van Schalkwyk et al., 2015). Phase 2 study design and assessment timeline are presented in Table 1.

**Table 1:** Phase 2 evaluation measures and timeline.

Outcomes/variables	Measure	Items	Timepoints	Report type
Treatment engagement	Yatchmenoff Engagement Scale (Yatchmenoff, 2005)	19	Baseline assessment, week 2, week 4, 3 -month follow-up	Self-report
Recovery	RAS-R (Corrigan et al., 2004; Salzer and Brusilovskiy, 2014)	24	Baseline assessment, week 2, week 4, 3 -month follow-up	Self-report
Internalized stigma	ISMI-9 (Boyd et al., 2014)	9	Baseline assessment, week 2, week 4, 3 -month follow-up	Self-report
Insight	VAGUS (Gerretsen et al., 2014)	10	Baseline assessment, week 2, week 4, 3 -month follow-up	Self-report
Psychotic symptoms	PANSS-6 (Kay et al., 1987; Østergaard et al., 2016)	6	Baseline assessment, 3 -month follow-up	Clinician-administered
Global illness severity	CGI-S (Guy, 1976)	1	Baseline assessment, 3 -month follow-up	Clinician-report
Global improvement of illness	CGI-I (Guy, 1976)	1	3-month follow-up	Clinician-report
Social and occupational functioning	SOFAS (Morosini et al., 2000)	1	Baseline assessment, 3 -month follow-up	Clinician-report
Substance use	WHO ASSIST (WHO ASSIST Working Group, 2002; Hides et al., 2009)	8	Baseline assessment, 3 -month follow-up	Clinician-administered
Alcohol, cannabis use disorder diagnosis	SCID-V (First et al., 2015)	11^a^	Baseline assessment, 3 -month follow-up	Clinician-administered
Adversity	TALE (Carr et al., 2018)	21	Baseline assessment	Self-report
Treatment attendance	Number of missed appointments	1	3-months pre-baseline, 3-months following participation completion	Administrative Record

### Phase 2: data analysis plan

A target sample size of 40 participants (20 per group) was determined *a priori* using G*Power (ANOVA: repeated measures, within-between interaction). The calculation assumed an effect size of *f* = 0.25, equivalent to Cohen’s d = 0.50 and corresponding to the smallest of the effects reported in the original Just Do You trial (Munson et al., 2022); α = 0.05; 95% power; two groups; four assessment points; and a correlation among repeated measures of 0.50. This indicated a required sample of 36 participants, which was rounded up to 40. No additional allowance for attrition was made. As a pilot evaluation, this study is powered to detect effects on the primary outcomes but is not powered for the exploratory subgroup analyses described above; non-significant findings should therefore not be interpreted as evidence of absence of effect. Primary outcomes (engagement, recovery, internalized stigma, and insight) will be analyzed using linear mixed-effects models to account for repeated measures and within-subject variation over time (Liu et al., 2012; West, 2009). Independent-samples t-tests will be used to assess baseline group differences in secondary outcomes. Identified significant between-group differences in these outcomes will lead to inclusion of these variables as covariates in multivariate analyses.

Secondary analyses of administrative outcomes will be modelled using generalized linear mixed-effects models with poisson or negative binomial distributions li. Where data are skewed or overdispersed, alternative models such as zero-inflated Poisson will be considered (Weaver et al., 2015). Subgroup analyses will evaluate differences in intervention effects based on referral source. These exploratory analyses will be interpreted descriptively given the pilot nature of the study, and will inform the design of a future, fully powered evaluation.

Mixed-effects models will use all available observations, allowing participants with incomplete data to be retained in analyses under a missing-at-random assumption without listwise deletion. Because engagement is the primary outcome of interest, missingness may not be fully at random: participants who disengage from services may also be less likely to complete follow-up assessments, and estimates may therefore be biased toward those who remain engaged in treatment. Administrative outcomes, including missed appointments, emergency department presentations, and hospitalizations, are extracted from clinical records and are therefore available irrespective of assessment completion, providing an indicator of engagement not subject to this form of attrition. Attrition will be reported in a CONSORT-style flow diagram.

## Results

### Phase 1: adaptation of the just do you intervention

#### Patient perspectives and adaptation themes

Consultation with patient partners yielded a range of feedback that directly informed revisions to the *Just Do You* intervention. Input and changes to the protocol correspond to four overarching themes: (1) fostering trust and supporting autonomy, (2) enhancing accessibility and relevance to early phase psychosis, (3) modifying session structure to enhance feasibility, and (4) supporting continuity beyond sessions. Below, we describe each theme and outline how it informed changes to the intervention protocol. Table 2 summarizes stakeholder feedback and resulting adaptations by theme, while Table 3 compares components of the original and adapted interventions.

**Table 2:** Adaptation process and results.

Original content/component	Stakeholder feedback	Resulting adaptation
Module 1: SAMHSA 12 principles of recovery	Language was too complex; too many items to engage with meaningfully	Replaced with SAMHSA 10-item working definition and visual recovery wheel; added discussion prompts *(Enhancing Feasibility)*
Module 1: peer support worker narrative	Valued recovery story; recommended emphasis be placed on challenges/setbacks overcome and long-term nature of recovery	Revised script to emphasize non-linear recovery, setbacks, and normalization of symptoms *(Enhancing Relevance)*
Module 1: videos of celebrities discussing mental health challenges and service use	Videos with others discussing and normalizing mental health challenges were seen as helpful. Consultants expressed concerns about the length of current videos and lack of psychosis-specific material.	Previous videos were replaced/augmented with brief, psychosis-specific videos tailored to participant experience *(Enhancing Relevance)* New videos were brief and segmented up when needed to discuss in order to enhance engagement. *(Enhancing Feasibility)*
Module 1: goal setting activity	Goal setting valued was valued; Consultants recommended including broader categories of goals (e.g., leisure) and facilitators provide increased support with goal formation and follow-up to match participants varied readiness and ability in early recovery.	Goal areas were expanded to recreation. Psychoeducation on SMART goals was added, and facilitators engaged participants in collaborative goal setting during sessions. *(Enhancing Relevance; Enhancing Feasibility)* Preliminary Goals were recorded on a handout to be shared with participants’ clinicians and family. *(Supporting Continuity)*
Module 1: discussion of trust in services and self-advocacy with healthcare providers	Consultants valued this section; recommended expanding discussion of past negative care experiences, barriers to trust, and normalizing mistrust. Consultants suggested adding concrete trust-building steps following discussion.	The trust exercise was revised informed by the CBT continuum technique. Participants rated past healthcare experiences on a 0–100 scale based on self-selected values to support reflection. *(Enhancing Relevance)* Participants and facilitators collaboratively identified opportunities for participants to build trust and practice self-advocacy with clinicians. *(Supporting Continuity)*
Module 1: knowledge of services and how they can help (Intro to CBT)	Consultants noted CBT was not widely available in the current context; Recommended focusing on describing services that are available and adding psychosis-specific psychoeducation in the intervention to reduce confusion and stigma.	Pamphlet outlining available clinic services was added. CBT content was replaced with psychosis-specific psychoeducation introduced in Session 1 focusing on normalizing symptoms and discussing hope for recovery. *(Enhancing Relevance)* Psychoeducation materials were offered in print or digital format for participants to review, share with loved ones, and discuss with clinicians. *(Supporting Continuity)*
Module 2: past adversity, causes of psychosis, and hope– music videos	Music video exercise seen as valuable for exploring adversity, psychosis, and hope; concerns raised about relevance of selected videos and number of videos being burdensome.	Revised exercise to have participants select three personally meaningful songs to guide discussion of adversity, psychosis, and recovery. *(Fostering Trust and Autonomy)* Exercise segmented with facilitator-led discussion between songs to enhance engagement and collaboration. *(Enhancing Feasibility)*
Module 2: past adversity, causes of psychosis, and hope– art exercise	Art exercise seen as helpful for exploring adversity and recovery; flexibility recommended as it may not suit all participants	Art exercises retained as optional; participants could choose art or verbal discussion based on preference*. (Fostering Trust and Autonomy)*
Module 2: peer support worker narrative, adversity, recovery, hope	Consultants recommended PSW share specific examples of overcoming engagement barriers and expand their role in this exercise.	PSW’s role expanded to include sharing recovery experiences during music and art exercises and complete exercises alongside participant. *(Fostering Trust and Autonomy)*
Module 2: intervention material review and wrap-up	Wrap-up discussion seen as helpful; consultants recommended ensuring topics could be revisited with clinicians or loved ones.	Participants received psychoeducation materials, goal-setting form, and service pamphlet; sessions ended with a collaborative discussion on applying material moving forward. (*Supporting Continuity*)
Overall protocol: general adaptations	Concern about cognitive overload and attentional challenges in session.	Materials were simplified; breaks offered; participants could choose to complete content over three shorter sessions. (*Enhancing feasibility*).
Overall protocol: general adaptations	While originally designed for group delivery, concerns were raised about feasibility for some participants early in treatment.	Participants given option of group or individual format based on preference. (*Fostering Trust and Autonomy; Enhancing Feasibility*)

Feedback and resulting adaptations were compiled from structured meeting minutes and subsequent stakeholder review.

**Table 3:** Comparison of components: Initial protocol and adapted protocol.

Module	Just Do You protocol	Adapted protocol	Change type
Module 1	SAHMSA 12 Principles of Recovery	SAMHSA 10 Principles (visual only)	Modified
	Peer Support Worker Narrative: Recovery	Peer Support Worker Narrative	Retained
	Celebrity Role Model Mental health Videos	Psychosis-specific Role Model videos	Replaced
	Goal Setting Activity	Modified SMART Goal Setting Activity	Modified
	Discussion of trust and barriers to engagement (e.g., prior negative healthcare experiences) +	Modified discussion of trust and barriers to engagement; Goal setting around self-advocacy	Modified
	Peer Support Worker Narrative: Trust.	Peer Support Worker Narrative: Trust (Focusing on providing concrete examples of barriers to engagement)	Modified
	Discussion of available clinic services.	Modified Discussion of available clinic services specific to NSEPP	Modified
	Intro to CBT + CBT Video		Removed
		Psychosis Formulation and psychoeducation	Added
	Debrief	Debrief with addition of take-home materials (psychoeducation, goal setting, and clinic pamphlet).	Modified
Module 2	Discussion of past adversity and causes of psychosis: Music Video Exercise with pre-approved songs.	Discussion of past adversity and causes of psychosis: Music Video Exercise with personally relevant songs.	Modified
	Discussion of past adversity and causes of psychosis: Art Exercise	Discussion of past adversity and causes of psychosis: Art Exercise (Optional) or continued verbal discussion.	Modified
	Peer support worker narrative: adversity, recovery, hope	Peer Support Worker Narrative: Adversity, Recovery, Hope + increased Peer Support Worker involvement throughout Module 2	Modified
	Debrief and wrap-up	Modified Debrief & Wrap-up with additional discussion around applying material discussed beyond sessions.	Modified

##### Fostering trust and supporting autonomy

Patient partners emphasized that trust in EIS is often compromised by prior healthcare experiences, especially those involving emergency departments, police, or involuntary hospitalization. Prior experiences were described as contributing to apprehension at the time of referral to EIS and influenced how participants engaged with providers. To support strong initial engagement in the brief intervention, patient partners highlighted the importance of fostering a welcoming and open therapeutic environment. Service users described that having an actively involved peer support worker (PSW) and emphasizing personal autonomy within sessions were especially important to facilitate a welcoming environment and therefore trust and engagement.

Based on this feedback, the role of the peer support worker (PSW) was expanded. In the adapted protocol, the PSW was encouraged to play a more active role in co-facilitating the intervention beyond discussing their lived experience at pre-specified points in sessions. This approach was viewed as less intimidating for new referrals and more conducive to building early trust and buy-in to sessions. In addition, in an attempt to broadly foster participant autonomy during delivery, several new elements were introduced to enhance participant autonomy: individuals can select whether to receive the intervention in a group or individual format; choose how session materials are provided (e.g., printed or digital); and select music for the second session from their own preferences, rather than a pre-approved list used in the original protocol.

##### Enhancing accessibility and relevance to early phase psychosis

While service users found the original *Just Do You* materials generally helpful, they felt that some content was not specific to the experiences of individuals with psychosis. In particular, patient partners noted that a lack of psychosis-specific examples and psychoeducation could limit individuals’ engagement with intervention materials, contribute to stigma, and limit understanding of symptoms and recovery.

To improve relevance, the adapted intervention includes new psychoeducation materials tailored to psychosis, which are presented at the beginning of Module 1. These materials aim to normalize experiences, reduce stigma, and offer hopeful, recovery-oriented messaging. They were written in accessible, non-pathologizing language, using terms familiar to individuals new to EIS.

In addition, new psychosis-specific videos replaced the general mental health content from the original intervention, and a program-specific brochure outlining all available services was prepared to ensure that materials were relevant to participants’ needs and clinical context. Patient partners emphasized the value of providing videos featuring individuals describing their personal experiences with specific symptoms of psychosis, challenges with engagement in services, and recovery journeys. Accordingly, videos were selected or developed based on stakeholder recommendations and included both publicly available materials and short clips created by the research team in collaboration with service users, who reviewed and approved the final selections. Content introducing CBT and emotional literacy in the original modules was removed, as CBT may not be offered to all participants at NSEPP (i.e., client-specific care).

##### Modifying session structure to enhance acceptability and feasibility

Stakeholders highlighted that individuals in early recovery from psychosis often experience cognitive, attentional, and motivational difficulties that can interfere with participation. As a result, simplifying content, making delivery more supportive, and pacing the intervention appropriately was considered essential.

In response, manual language was revised to be more conversational and accessible with academic jargon being removed and replaced with lay-friendly language. A newer version of SAMHSA’s working definition of recovery was included (Ellison et al., 2018), which is provided in more accessible language, and all multimedia materials were shortened and segmented to improve focus and engagement and allow participants more opportunity to comment. Facilitators were encouraged to remain flexible in pacing and, where needed, to provide breaks throughout delivery or deliver the content over three shorter sessions rather than two.

The original goal-setting activity was also expanded. New categories, such as leisure and recreation, were added based on participant input. To support individuals who may struggle with goal formulation due to cognitive symptoms or negative symptoms of psychosis, the concept of SMART (Specific, Measurable, Achievable, Realistic, Time-Limited) (Fenn and Byrne, 2013), goals was introduced and used in session to guide collaborative goal-setting with facilitators.

##### Supporting continuity beyond sessions

Stakeholders expressed a strong desire to involve loved ones and primary clinicians at EIS in the recovery process and to maintain continuity after the intervention ended. Although direct family or clinician involvement in sessions was not feasible due to confidentiality restrictions, participants wanted to be able to share what they had learned and discussed during sessions with their broader support network. To support this, participants are now given copies of their worksheets and psychoeducational materials, which they can optionally share with family or support persons. Participants are also encouraged to build on intervention topics with their primary clinicians. With participant consent, relevant clinical staff may receive summaries of the goals discussed and planned next steps.

Stakeholders also identified a lack of knowledge around service availability as this is a patient population new to psychiatric care in many cases. To address gaps in service awareness, a new locally tailored information booklet was added to the intervention. This resource outlines available clinical and community supports at NSEPP and is introduced during the first session to promote service navigation and follow-up beyond the intervention.

## Discussion and implications

This study aimed to adapt an existing brief intervention to support engagement for use within an early intervention service (EIS) for psychosis. Through a series of stakeholder consultations, informed by the Formative Method for Adapting Psychotherapy (FMAP) and the Psychotherapy Adaptation and Modification Framework (PAMF), a provisional adapted version of the intervention was developed and is now being delivered for evaluation. Adaptations were guided by both existing literature and stakeholder input and changes made broadly correspond to adaptations across 4 core domains: 1. Fostering Trust and Supporting Autonomy, 2. Enhancing Accessibility and Relevance to Early Phase Psychosis, 3. Modifying Session Structure to Enhance Acceptability and Feasibility, and 4. Supporting Continuity Beyond Sessions.

The adapted intervention was designed to prioritize flexibility, simplicity, and autonomy. It is intended to increase patient choice in content delivery and session structure, while incorporating psychosis-specific psychoeducation and materials. Importantly, the peer support worker role was expanded with the aim of facilitating trust and improving engagement. These changes align with our group’s prior qualitative research, which underscored the importance of a collaborative therapeutic relationship that prioritizes patient autonomy, transparency, and a shared understanding of patients symptoms and treatment (Senger et al., 2025). These factors were seen as especially important for fostering initial engagement in EIS, particularly given that individuals in this population often enter care following coercive, distressing, or confusing healthcare experiences (Senger et al., 2025).

Our findings are consistent with broader research on adapting psychosocial interventions for individuals with psychosis (Lakeman, 2006; Langman-Levy et al., 2023; Lecomte et al., 2017). Prior literature highlights the importance of flexibility in session pacing and structure, as well as the use of accessible language, to accommodate common cognitive challenges and negative symptoms in this population. Additionally, while trust-building, normalization of symptoms, and provision of relevant recovery-oriented psychoeducation are essential to all therapeutic relationships, prior research suggests these elements require particular emphasis in early psychosis care. Mistrust, stigma, and uncertainty about one’s experiences can pose substantial barriers to engagement, making these factors especially critical for facilitating engagement early in treatment (Lakeman, 2006; Langman-Levy et al., 2023; Lecomte et al., 2017).

Informed by this work, our revised protocol includes additional psychosis specific introductory psycho-education material, simplified language, and the option to pace the intervention over three shorter sessions if needed. While family involvement in-session was not feasible, participants now receive take-home materials for loved ones and have the option to share intervention content with clinicians, supporting continuity of care across the participant’s broader support network.

The adaptation process employed an iterative approach combining top-down (literature- and theory-informed) and bottom-up (stakeholder-informed) elements. This method was intended to ensure that the revised intervention incorporated evidence-informed modifications while also reflecting the preferences of service users. Notably, there was strong convergence between stakeholder recommendations and existing literature on useful adaptations for psychotherapy with individuals experiencing psychosis. This alignment may support the relevance and potential utility of the adapted protocol (Lakeman, 2006; Langman-Levy et al., 2023; Lecomte et al., 2017).

Despite these strengths, the adaptation process had several limitations. First, consultation involved a small number of stakeholders (*n* = 7), with all service users in this group currently having strong engagement in treatment (*n* = 4). Their feedback may not reflect the full range of perspectives, particularly those of individuals with precarious engagement or those who have disengaged entirely, who may stand to benefit most from engagement-focused interventions. Future work should prioritize the inclusion of individuals with more diverse experiences of service engagement.

Second, while family involvement was frequently recommended by stakeholders, the study was unable to include loved ones directly in the intervention. This limitation reflects a broader challenge in EIS: although family support has been identified as a facilitator engagement in treatment, the best ways to incorporate loved ones while respecting patient autonomy and privacy remain unclear (Lecomte et al., 2017). Future work should explore structured models of family involvement that strike this balance.

Third, as consultation meetings were not audio-recorded or transcribed, the adaptation process relied on structured minutes rather than verbatim records. While minutes were circulated to stakeholders to confirm accuracy, this approach may not have captured the full nuance of discussions.

Fourth, because disengagement from services and non-completion of study assessments may share common causes, missingness in self-reported outcomes may not be fully at random. Administrative indicators of engagement are not subject to this limitation, but self-reported outcomes will be interpreted with this possibility in mind.

The adapted *Just Do You* protocol was implemented and evaluated in fulfilment of Phase 2 of the trial. Results from this trial will be reported separately and will provide preliminary evidence regarding the intervention’s effectiveness in improving engagement among young adults newly referred to early intervention services (EIS) during a critical period of their recovery. If shown to be effective, the adapted intervention’s brief and flexible format (two 90–120-min sessions delivered individually or in groups) may support its feasibility for implementation across diverse clinical settings. Though initially designed by clinicians with doctoral-level training, it is intended to be feasibly implemented by a broad range of providers (e.g., nurses, occupational therapists, social workers) with appropriate training, which may further support scalability and potential cost-effectiveness of the intervention (Munson et al., 2020, 2022).

As previously discussed, early and sustained engagement in treatment is fundamental to the efficacy of EIS. Despite this, disengagement remains a persistent challenge, and few interventions have been developed to address it. Most existing research has focused on describing disengagement trends and barriers and facilitators of engagement or discussing a need for system level change in how services are delivered, rather than developing engagement-enhancing strategies. While systems change is clearly needed, modifications to the way services are to be delivered are likely to require significant coordination and funding. This study represents an early attempt to develop and evaluate a theory and stakeholder-informed intervention that directly targets cognitive and affective barriers to engagement, such as, hopelessness, stigma, mistrust, and negative beliefs about treatment and providers.

## Conclusion

This study describes the adaptation and early implementation of a brief, engagement-focused intervention for individuals accessing EIS for psychosis. If found to be effective, the intervention may provide a scalable, low-cost approach to improving early engagement in EIS, thereby potentially supporting improved long-term outcomes. Regardless of the trial’s outcome, this work contributes meaningfully to the limited literature on interventions addressing barriers to engagement and may serve as a foundation for future research in this area.
